# Comparison study between mesoporous silica nanoscale microsphere and active carbon used as the matrix of shape-stabilized phase change material

**DOI:** 10.1038/s41598-019-52553-3

**Published:** 2019-11-05

**Authors:** Zijun Zhang, Jingxing Wang, Xi Tang, Yi Liu, Zhi Han, Yan Chen

**Affiliations:** 1 School of Port and Transportation Engineering, Zhejiang Ocean University, Zhoushan, 316022 China; 2 School of Foreign Languages, Zhejiang Ocean University, Zhoushan, 316022 China

**Keywords:** Energy storage, Energy

## Abstract

Mesoporous silica nanoscale microsphere (MSNM), with a special morphology, high porosity, large pore volume and specific surface area, was successfully prepared and used as the matrix material of lauric acid (LA) to prepare a favorable shape-stabilized phase change material (LA/MSNM). The porous network structure of MSNM is effective to prevent the leakage and enhance the thermal stability of LA/MSNM. For comparison, shape-stabilized phase change material of LA/AC, which contained commercially purchased active carbon (AC) and LA, was prepared by the same method. Characterizations of LA/MSNM and LA/AC, such as chemical properties, structure, thermal properties and crystallization properties were studied. The mechanisms of interaction between LA molecules and MSNM or AC were explicated. The results of TGA test showed that the LA/MSNM and LA/AC had superior thermal stability, and however, the melting and solidification enthalpies of LA/MSNM were much higher than that of LA/AC, which was attributed that the loading capacity of MSNM was better than that of LA/AC. All of the study results demonstrated that the mesoporous silica nanoscale microspheres of MSNM synthesized in this study possessed the potential for practical applications as a suitable supporter of organic phase change materials.

## Introduction

Energy conservation and environmental protection have become a worldwide concern with the continuous development of human economic society and mass consumption of energy resources. Utilizing energy storage materials to maintain the balance between the world’s growing energy demand and supply can effectively improve the efficiency of energy development and utilization in order to achieve the objective of energy conservation and environmental protection^1,2^.

Phase change materials (PCMs) can absorb or release heat during the phase change process, and then adjust the ambient temperature^3^. PCMs have the merits of high latent heat, high thermal energy storage density and constant temperature during absorbing and releasing heat^4^. PCMs have been diffusely applied in energy saving for buildings, solar energy utilization, recovery of industrial waste heat, thermal insulation of textile and clothing, air-conditioning systems, thermal management and so on^5,6^. Additionally, in order to manage the heat produced by electronic equipment such as heavy circuit chips, Li-ion batteries and computers, and prolonged their service life, PCMs could be used for cooling of electronic devices because of their high latent heat, easy availability and low costs^7^. Moreover, for the purpose of improving the heat radiation efficiency of heat sinks, PCMs are also commonly used as coolant to cool down heat sinks, which could enhance their heat transfer rates^8,9^. Based on the composition of PCMs, they can be categorized into organic and inorganic PCMs^10^. Compared with inorganic PCMs, organic PCMs have many advantages, such as good thermal stability, stable chemical properties, less corrosiveness, small volume change before and after phase change process, almost no phase separation, and so on^11,12^. Therefore, organic PCMs have great potential for practical applications in latent heat storage. Fatty acid is one of the most attractive organic PCMs for the reason that it has many advantages, such as high latent heat, small undercooling, good thermal and chemical stability, non-toxic, non-corrosive and so on^13^. These advantages make it outstanding in energy storage applications. In addition, fatty acids are rich in sources and are easy to extract^14^ from vegetable oils and fats. However, when the fatty acid is directly used as the phase change material (PCM), it still has some shortcomings: liquid leakage is easy to occur in solid-liquid phase change process; low thermal conductivity limits the thermal conduction of composite PCMs.

In order to solve the above two problems, porous materials, such as porous silica, porous carbon materials, porous metal oxides and so on, are introduced into PCMs to prepare shape-stabilized PCMs^15^. Chen *et al*. utilized lauric acid and activated carbon to synthesize composite PCMs^16^. Their experimental results demonstrated that the composite PCM could prevent the leakage of molten lauric acid. Mehrali *et al*. prepared stearic acid/carbon nanosphere PCMs at 35 °C. The thermal conductivity of the shape-stabilized PCM was increased by about 105% than that of the pure stearic acid^17^. However, when the porous material is used to synthesize shape-stabilized PCM, its pore size will affect the phase change behavior of the organic PCM. If the average pore size is too small, the molecular motion of the organic PCM will be held back, which will affect their capacity of latent heat storage^18^. Contrarily, if the pore size is too large, there will not be enough capillary force to retain the liquid PCM, which will lead to leakage^19^. In the research of Tian *et al*., polyethylene glycols (PEG) were embedded in silica gels (SG). They found that PEG/SG composites exhibited thermal properties correlated with the pore size of SG^20^. Mesoporous silica, as the matrix material of shape-stabilized PCMs, can avoid the leakage of organic PCMs in the phase change process, and improve their thermal conductivity. More importantly, the high porosity of mesoporous materials can increase the loading amount of organic PCMs and then the phase change enthalpy of shape-stabilized PCMs could be improved. Qian *et al*. prepared mesoporous silica by simple self-assembly method, and then utilized it as the carrier of polyethylene glycol to prepare shape-stabilized PCM^19^. Kadoono *et al*. utilized silicon-based molecular sieves (SBA-15) with nano-ordered pore structures as the containers for stabilizing fatty acids, and the ordered pore structures could increase the heat recovery rate during the phase change process^21^. Zhang *et al*. prepared graphene-based mesoporous silicon sheet (GS) as a new mesoporous material for the adsorption of polyethylene glycol to synthesize a new type of phase change composite material, and the problems of leakage and low conductivity were solved by GS, and there was no phase separation in the phase change process^22^.

In this study, mesoporous silica nanoscale microsphere (MSNM) was synthesized. It has the merits of high porosity, large specific surface area, large mechanical strength and good thermal stability. And then, the MSNM was used as the matrix material of organic lauric acid to prepare shape-stabilized PCM (LA/MSNM). For comparison, shape-stabilized PCM of LA/AC, which contained lauric acid and commercially purchased active carbon (AC), was prepared by the same method. The characterizations of LA/MSNM and LA/AC composite shape-stabilized PCM were studied, and the results manifested that the thermal properties of LA/MSNM were better than that of LA/AC, and the MSNM could be used as a potential supporter of organic PCM in the practical application of thermal energy storage.

## Experimental

### Materials

Triblock poly(ethylene oxide)-poly(propylene oxide)-poly(ethylene oxide) copolymer (P123) and mesitylene were obtained from Sigma-Aldrich. Tetraethyl-orthosilicate (TEOS) was purchased from Changzhou Wuhuan Synthetic Materials Co., Ltd. Hydrochloride was purchased from Sinopharm Chemical Reagent Co., LTD (Shanghai, China). Ethanol was purchased from Tianjin Jiangtian Chemical Technology Co., Ltd. Lauric acid (LA) and activated carbon (AC) were obtained from Meryer Chemical Technology Co., Ltd., China.

### Synthesis of MSNM

MSNM was synthesized according to the method that Jun *et al*. reported^23^. The specific steps were as follows: P123 (4.0 g) was firstly added into the beaker, and then hydrochloric acid (150 mL 1.6 M) was added. The mixture above was kept at 20 (°C) in constant temperature water bath and was stirred for 3 (h), and then mesitylene (2.0 g) was put into it, continuing stirring for 5 (h), until the formation of emulsion. After that the bath temperature was raised to 40 (°C), and then TEOS (9.1 mL) was added with rapidly stirring, and then the solution was stirred for 20 (h). After the reaction was completed, the reaction solution was transferred into a hydrothermal reactor with PTFE lining, and crystallized at 100 (°C) for 24 (h). After crystallization, the suspension was centrifuged and washed with distilled water for three times, and then it was dried at 50 (°C) for 12 (h)^24^. After drying, the product was baked at 500 (°C) for 6 (h) in a muffle furnace at the heating rate of 1 (°C/min). Finally, the mesoporous silica nanoscale microspheres (MSNM) was obtained.

### Synthesis of LA/MSNM

**Figure 1:**
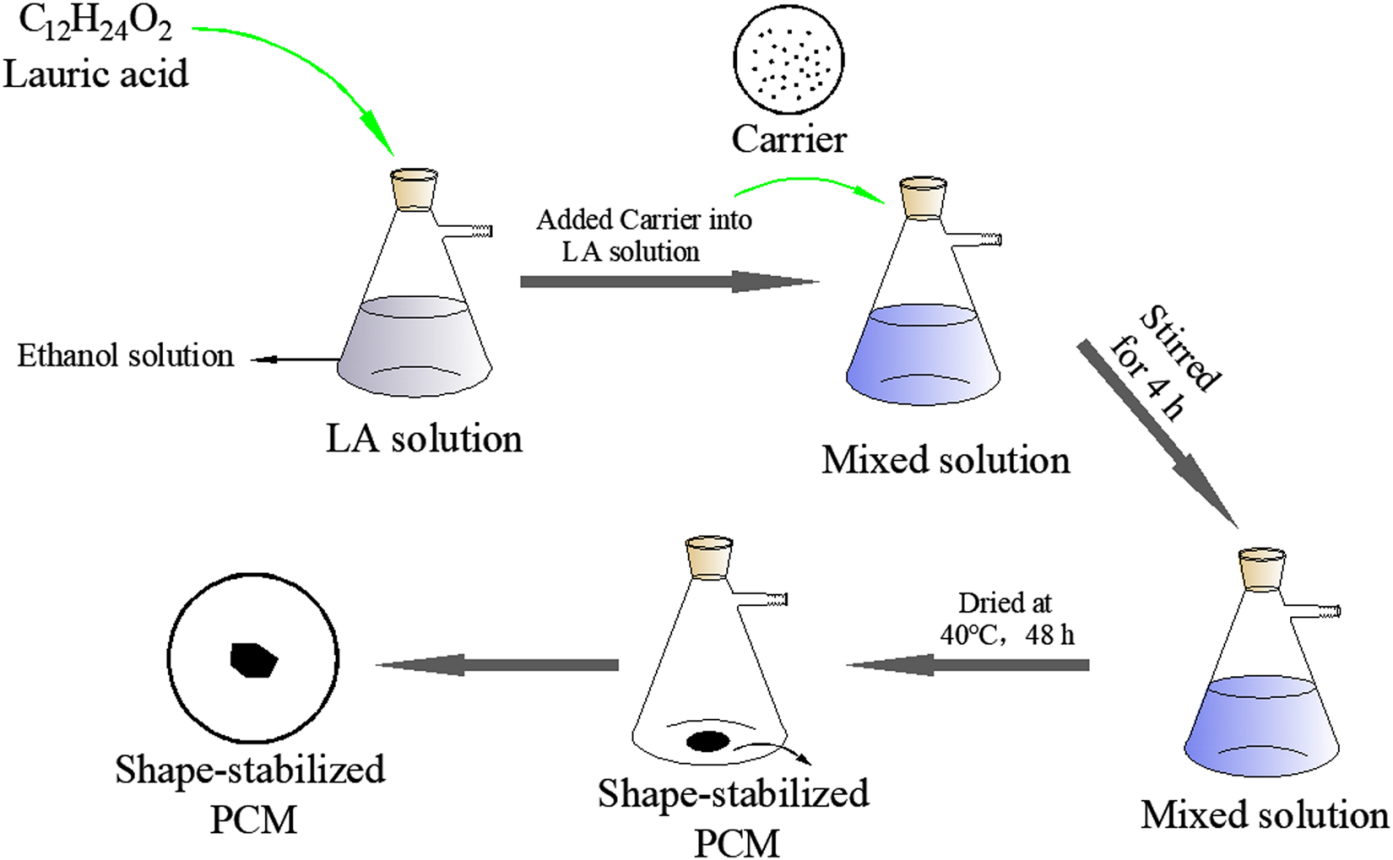
Schematic diagram of LA/AC and LA/MSNM synthesization.

Figure 1 shows the schematic diagram of LA/AC and LA/MSNM synthesization. The LA/MSNM was prepared by physical blending and impregnation method. The typical preparation process was as follows: firstly, a certain amount of LA was dissolved in 15 (mL) ethanol. 0.5 (g) MSNM was then added into the solution and stirred for 4 (h). Finally, LA/MSNM composite PCMs were prepared by drying the mixed liquor at 40 (°C) for 48 (h). For comparison, LA/AC composite PCMs were synthesized by the same method with commercially purchased active carbon (AC) as the carrier and lauric acid as the core material. In order to test the maximum loading amount of LA in the shape-stabilized PCMs, the LA/MSNM and LA/AC composites with LA content of 40 wt%, 50 wt% and 60 wt% were prepared respectively, and then the composites were maintained at 55 (°C) to tests their stability. It was found that there was no leakage observed for 60 wt% LA/MSNM and 50 wt% LA/AC. Thus, the 60 wt% LA/MSNM and 50 wt% LA/AC were mainly studied in this paper.

### Characterization of LA composite shape-stabilized PCM

The microstructure and morphology were analyzed by S-4800 scanning electron microscope (SEM). The microstructure of MSNM was studied by transmission electron microscopy (TEM) of JEM-2100F, Japan. N_2_ adsorption/desorption isotherms of mesoporous matrix materials (AC, MSNM) were measured by ASAP 2020 physical adsorption apparatus. The infrared absorption spectra (FT-IR) of the raw materials and the composite PCMs were measured by Nicolet 6700 Fourier transform infrared spectrometer. The crystal structure of the composite PCMs were analyzed by D/MAX-2500 X-ray diffractometer. The phase change temperature and latent heat of LA and LA composite PCMs were measured by DSC200F3 Netzsch differential scanning calorimeter, Germany. The samples were heated and cooled at a temperature of 0~100 (°C) in a high purity atmosphere at the rate of 10 (°C/min). The thermogravimetric analysis of pure LA and the composite PCMs was carried out under protection of nitrogen atmosphere with STA-449F3 Netzsch thermogravimetric analyzer, Germany, and the temperature rising range was 10~550 (°C), and the heating rate was 10 (°C/min).

## Results and Discussion

### Microstructure of MSNM and AC

**Figure 2:**
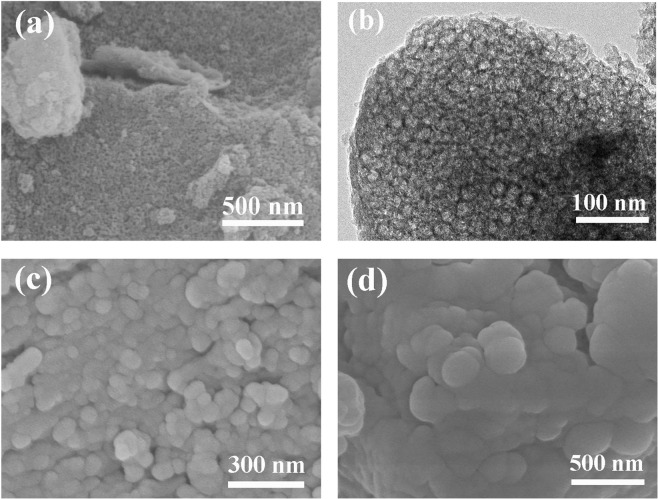
SEM image of (**a**) MSNM, TEM image of (**b**) MSNM and SEM images of (**c**,**d**) LA/MSNM.

Figure 2(a,b) show the SEM and TEM images of MSNM, respectively. Figure 2(a) shows that MSNM was composed of many nanoscale spherical particles, which were bound together to form micron-sized particles with secondary structure. From Fig. 2(b), it is clear that MSNM presented a spherical structure, and the channel curvature had outward exposure. Figure 2(c,d) are the SEM images of LA/MSNM composite PCMs. From the images, LA was firmly adsorbed into the porous network structure of MSNM, which was due to the action of surface tension and capillary force between the LA molecules and MSNM. In addition, due to the support of MSNM network skeleton, the liquid leakage problem of LA/MSNM was effectively overcome in the phase change process.

**Figure 3:**
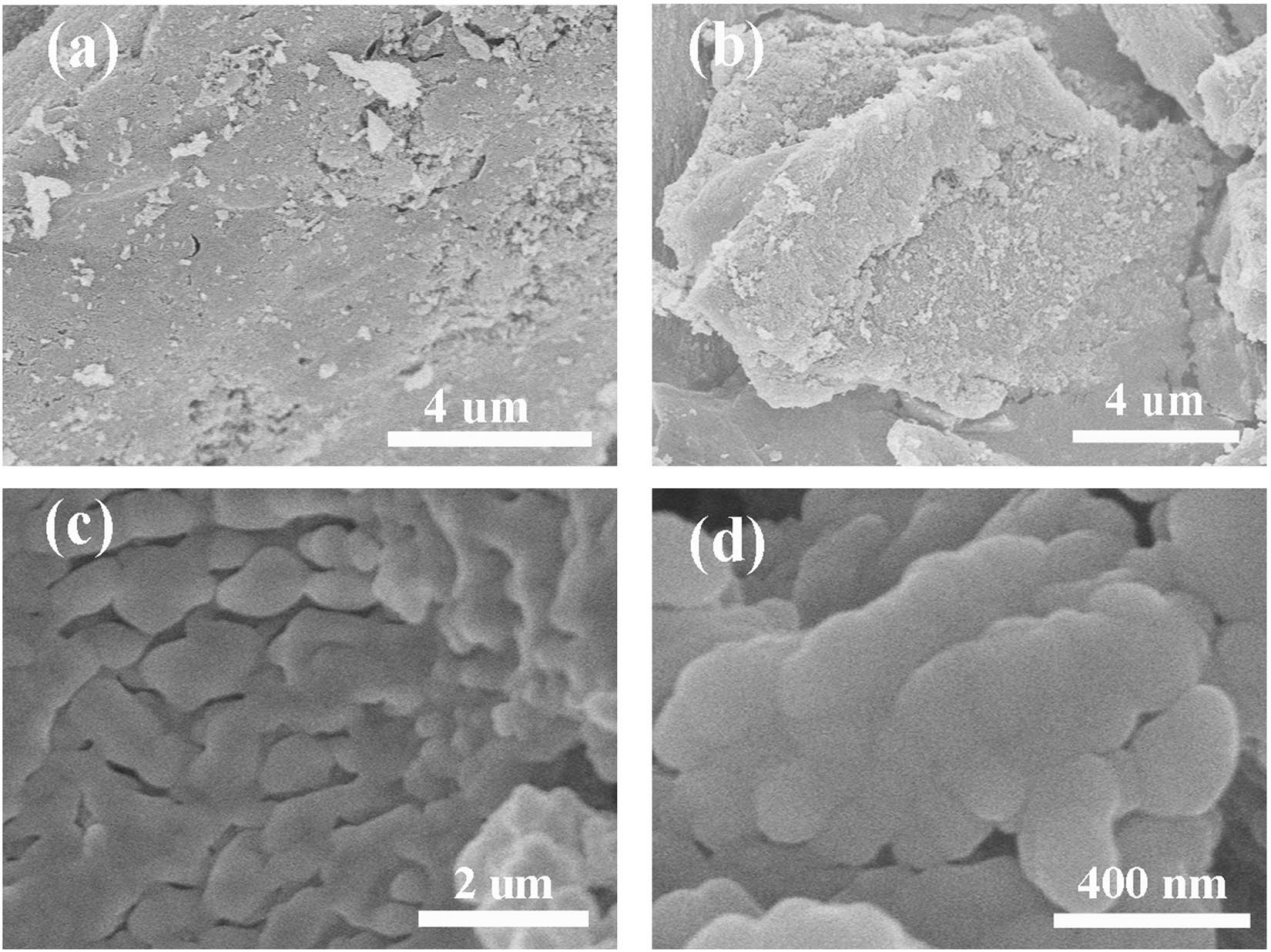
SEM images of (**a**,**b**) AC and (**c**,**d**) LA/AC.

Figure 3(a,b) are the SEM images of AC, and Fig. 3(c,d) are the SEM images of LA/AC composite PCMs. It could be observed that activated carbon had a rough surface and many carbon particles. From the SEM images of LA/AC, it could be observed that a large amount of LA was firmly adsorbed on the surface of AC, and the AC played the role of encapsulation and solved the problem of liquid leakage of the pure LA in the phase change process.

### N_2_ adsorption-desorption

**Figure 4:**
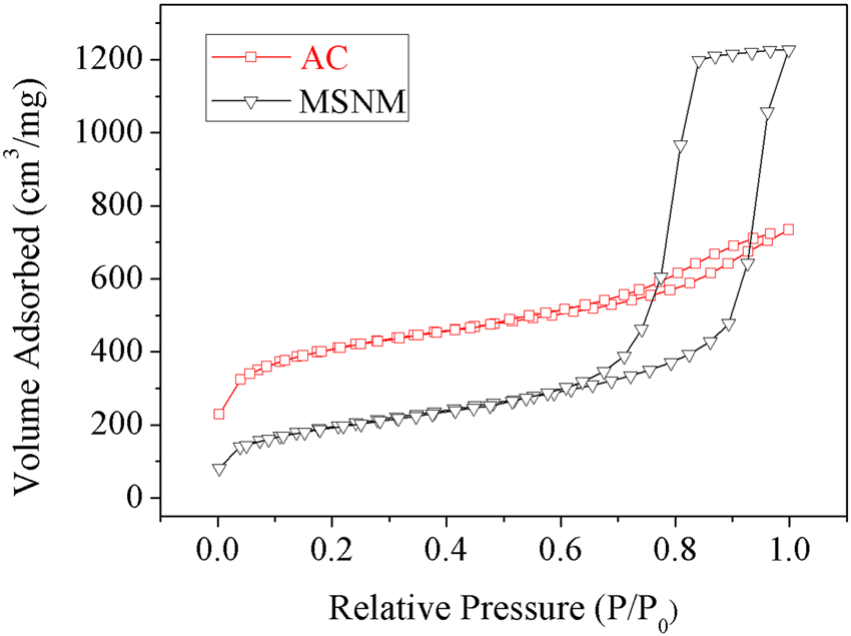
N_2_ adsorption-desorption isotherms of the MSNM and AC.

The adsorption/desorption isotherms of MSNM and AC are shown in Fig. 4. The adsorption equilibrium isotherms of MSNM and AC were typical type VI isotherms with H2 hysteresis loops, which were consistent with their mesoporous structures^25,26^. The BET specific surface area of MSNM was 690.7 (m^2^/g), and the BJH adsorption cumulative pore volume and pore diameter were 1.97 (cm^3^/g) and 10.3 (nm) respectively. The BET specific surface area of AC was 1358.2 (m^2^/g), and the BJH adsorption cumulative pore volume and pore diameter were 0.556 (cm^3^/g) and 3.20 (nm), respectively. Compared with AC, MSNM had a larger pore size and pore volume, which could be conducive to the smooth entry of LA molecules into the internal channels of MSNM, and also could improve the adsorption rate of LA by MSNM^27^.

### FT-IR analysis

**Figure 5:**
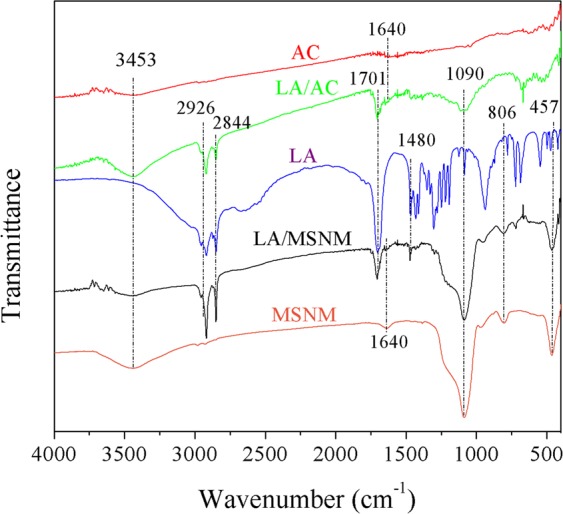
FT-IR spectra of LA, AC, MSNM, LA/AC and LA/MSNM.

Figure 5 shows the infrared spectra of pure LA, MSNM, LA/MSNM composites, AC and LA/AC composites. The absorption peaks of pure LA at 2926 (cm^11^) and 2844 (cm^−1^) were caused by the asymmetrical and symmetrical stretching vibrations of -CH_2_, respectively^28^. The peaks at 1701 (cm^−1^) and 1480 (cm^−1^) belonged to the C=O stretching vibration^29^. In MSNM spectrum, the asymmetric vibration peak of -OH and the bending vibration peak of H-O-H were at 3453 (cm^−1^) and 1640 (cm^−1^) respectively^30^. The peak at 1090 (cm^−1^) was caused by the asymmetric stretching vibration of Si-O-Si skeleton^31,32^. The peaks at 806 (cm^−1^) and 457 (cm^−1^) were attributed to Si-O bond stretching vibration and tetrahedral Si-O bending vibration^12,30^. In the spectrum of LA/AC, two characteristic peaks at 3453 (cm^−1^) and 1640 (cm^−1^) were observed because of the vibration of -OH. In addition, in the spectrum of LA/MSNM, the absorption peaks at 2926 (cm^−1^) and 2844 (cm^−1^) were ascribed to the stretching vibration of the C-H, and the absorption peak at 1701 (cm^−1^) was ascribed to the stretching vibration of C=O. Compared with the infrared spectra of LA, MSNM, LA/MSNM, AC and LA/AC, the infrared spectra of LA/MSNM and LA/AC composite PCMs were basically superimposed by the characteristic peaks of each component without new characteristic absorption peaks. It was proved that the interaction between the LA and the matrix of MSNM or AC was physical chimerism, and the chemical structure of the phase change components did not change^33^.

### XRD analysis

**Figure 6:**
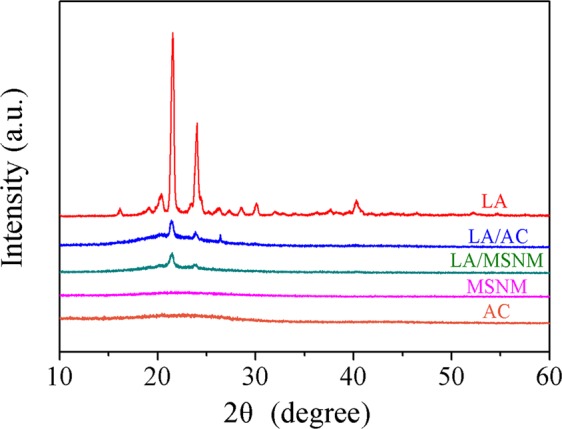
XRD patterns of LA, AC, MSNM, LA/AC and LA/MSNM.

Figure 6 is the X-ray diffraction patterns of pure LA, MSNM, AC, LA/AC and LA/MSNM composite PCMs. It can be seen from the X-ray diffraction patterns that pure LA had characteristic diffraction peaks at 21.7° and 23.8°, and the peak intensity was very high, indicating that it had good crystallization performance^34^. The MSNM diffraction pattern was dispersive, indicating that it was not crystal structure^35^. In addition, by observing the diffraction patterns of LA/MSNM, it is observed that the peak position of the characteristic diffraction peaks of the composites was roughly the same as that of the pure LA, which indicated that the crystalline morphology of LA in the composites had not changed. However, the peak strength of the XRD pattern for LA/MSNM decreased to a certain extent, which suggested that the crystalline properties of the composites were reduced due to the binding of inorganic matrix materials^35,36^. In addition, after the formation of LA/AC composites, the diffraction pattern of LA/AC were similar to that of LA, indicating that the polymer structure had not changed, but the absorption intensity had obviously changed. This was because LA was restricted by AC and its crystallization property declined, resulting in the decrease of the intensity of the crystalline diffraction peaks.

### Thermal properties of LA/MSNM and LA/AC

**Figure 7:**
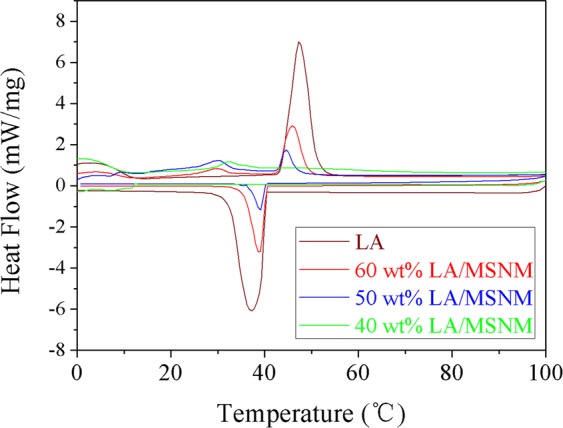
DSC curves of pure LA and LA/MSNM with different content of LA.

**Figure 8:**
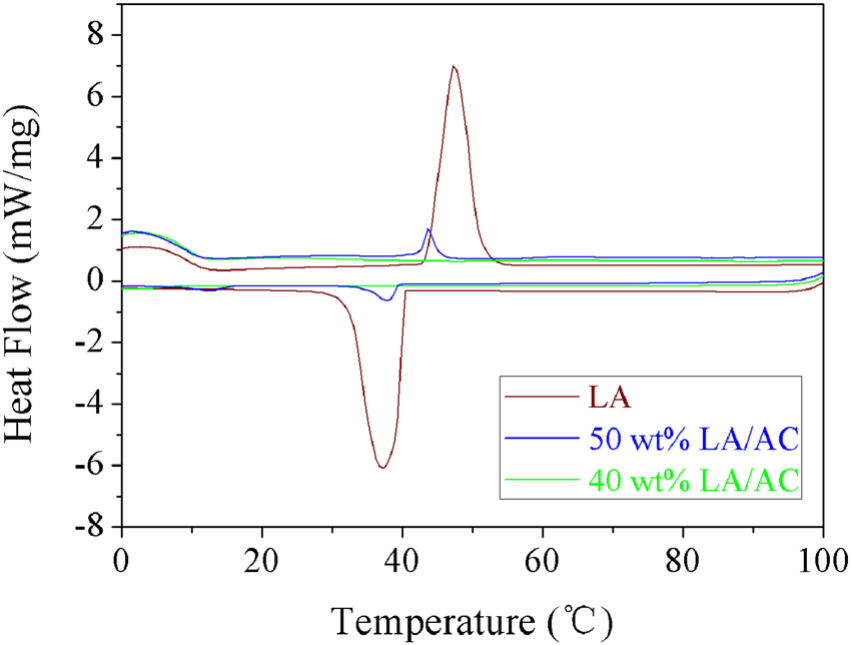
DSC curves of pure LA and LA/AC with different content of LA.

Figure 7 shows the DSC curves of pure LA and LA/MSNM composite PCMs with different LA contents. Table 1 shows the phase transition temperature and phase change enthalpy data of the samples. From Fig. 7, it could be seen that with the increase of LA content, the phase change enthalpy corresponding to LA/MSNM also increased, and the phase change temperature and the peak temperature changed slightly. With the increase of LA content, the phase change enthalpy corresponding to LA/MSNM also increased because the latent heat of the composite materials was induced by the phase change of lauric acid. Besides, the changes of phase change temperature and peak temperature were attributed to that the properties of heat storage particles were different from those in their accumulation state caused by surface tension^37^. In addition, it can be seen from Fig. 7 and Table 1 that there was an endothermic peak (T = 47.2 °C) during the heating process, an exothermic peak (T = 37.8 °C) in the cooling process, and the melting and solidification enthalpies of pure lauric acid were 174.7 (J/g) and 187.5 (J/g). The melting and solidification enthalpies of 60 wt% LA/MSNM composite PCM were 57.2 (J/g) and 58.8 (J/g), respectively, which were less than their theoretical enthalpy values, and this was mainly due to the interference of mesoporous materials on the crystallinity of lauric acid, which could be confirmed by XRD test results. And when the content of lauric acid decreased, the effect on the crystallinity of LA molecules was more significant, which led to the lower enthalpy of phase transition in composite phase transformation process. When the content of lauric acid decreased to a certain level (for example, 40 wt% LA/MSNM), the crystallization of lauric acid was completely limited, and the enthalpy of phase transformation of the composites was zero^36,38^. For comparison, differential thermal analysis of LA/AC composites was also carried out, and the matrix of AC was commercially purchased. The results are exhibited in Fig. 8 and Table 1. It could be seen from Fig. 8 that the peak area of 50 wt% LA/AC was much smaller than that of pure LA. In addition, the melting and solidification enthalpies of 50 wt% LA/AC were 13.4 (J/g) and 10.0 (J/g), respectively, which were much smaller than the theoretical values. The capillary effect of carbon nanochannels and van der waals force restrained the crystallization behavior of core materials, resulting in the decrease of LA crystallinity and phase transition enthalpy^39^.

**Table 1:** Comparison of thermal properties of the investigated materials based on DSC.

Sample name	Endothermic process				Exothermic process			
	T_start_ (°C)	T_end_ (°C)	T_peak_ (°C)	Melting enthalpy (J/g)	T_start_ (°C)	T_end_ (°C)	T_peak_ (°C)	Solidification enthalpy (J/g)
Pure LA	43.5	51.3	47.2	174.7	41.2	32.5	37.2	187.5
40 wt% LA/MSNM	—	—	—	—	—	—	—	—
50 wt% LA/MSNM	42.1	47.2	43.6	17.1	41.5	34.1	37.8	18.3
60 wt% LA/MSNM	42.6	48	44.7	57.2	41.6	33	39.3	58.8
40 wt% LA/AC	—	—	—	—	—	—	—	—
50 wt% LA/AC	39.0	46.8	43.8	13.4	39.1	33.5	37.1	10.0

Additionally, the melting and solidification enthalpies of LA/MSNM composite PCM were higher than that of the LA/AC composite PCM. Because the pore size of MSNM is larger than that of AC, not only the loading amount of LA on MSNM could be increased, but also the crystallization behavior of LA molecular in PCMs could be improved. Therefore, the enthalpy of LA/MSNM was higher than that of LA/AC.

**Figure 9:**
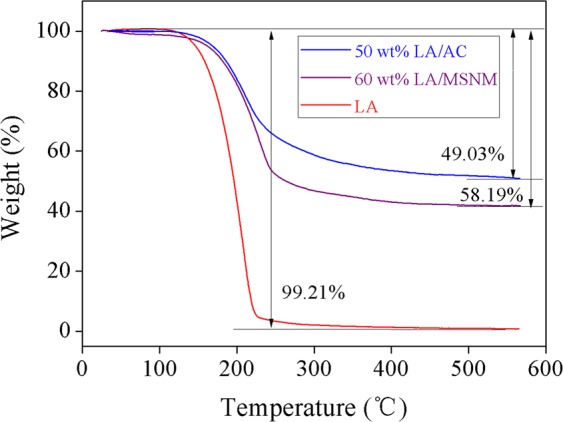
TGA curves of pure LA, 50 wt% LA/AC and 60 wt% LA/MSNM.

Figure 9 shows the TGA thermograms of the pure LA, 60 wt% LA/MSNM and 50 wt% LA/AC from 0 to 600 (°C). It could be seen from the figure that the initial weight loss temperature of pure LA was about 120 (°C), and the temperature range from 130 to 230 (°C) was its main weightlessness interval, which was attributed to the pyrolysis of LA, and the weight loss rate of LA at 230 (°C) was 95.61%. The LA/MSNM PCMs exhibited slight weight loss below 140 (°C), which was due to the desorption of adsorbed water from the physical force at high temperature. The weight loss interval of LA/MSNM was in the range of 140~250 (°C). The results revealed that the initial weight loss temperature of LA in the composite materials moved afterwards by about 10 (°C), and the weight loss interval was also prolonged, indicating that MSNM hindered the pyrolysis of LA. LA in the 60 wt% LA/MSNM was pyrolyzed completely at about 510 (°C), and the weight loss rate was 58.19%, which was almost consistent with that of 60% in the preparation experiment. The remaining part might be some high temperature resistant impurities. For comparison, the thermal stability of LA/AC prepared with commercially purchased activated carbon as the supporter of LA were tested and analyzed under the same experimental conditions. According to Fig. 9, there was only one weight loss step on the TGA curve of 50% LA/AC composite, and it had no thermal decomposition reaction and no obvious mass loss below 143 (°C), which indicated that 50% LA/AC had good thermal stability below 143 (°C). However, when the temperature reached 143 (°C), the decomposition reaction of LA components in 50% LA/AC began to take place until the decomposition reaction was almost over at about 520 (°C), and the residual mass fraction was 50.97%. Compared with pure LA, the weightlessness temperature of the two shape-stabilized PCMs was higher and the weightlessness area was wider, which illustrated that LA/MSNM and LA/AC composite materials had excellent thermal stability^40^.

### Comparison of LA/MSNM with other shape-stabilized PCMs

The latent heat of 60 wt% LA/MSNM was compared with other shape-stabilized PCMs also developed by physical adsorption through compounding LA with supporting materials, and the results are exhibited in Table 2. It was clear that the thermal storage capacity of LA/MSNM was competitive and favorable, though the melting and solidification enthalpies of LA/MSNM were not the highest. Besides, LA/MSNM had superior thermal stability and reliability, and the synthetic method of the LA/MSNM possessed the advantage of easy operation. Consequently, LA/MSNM possessed high potential in practical applications for thermal energy storage.

**Table 2:** Comparison of LA/MSNM with other shape-stabilized PCMs.

Sample name	Endothermic process		Exothermic process		References
	Melting temperature (°C)	Melting enthalpy (J/g)	Solidification temperature (°C)	Solidification enthalpy (J/g)	
60 wt% LA/MSNM	42.60	57.2	41.60	58.80	This work
LA/AC	43.87	32.45	41.67	30.08	Sajid *et al*.^16^
LA/diatomite	39.50	30.4	38.10	26.8	Konuklu *et al*.^41^
LA/polyethylene terephthalate	46.00	62.9	38.80	41.5	Wen *et al*.^42^
LA/polyethylene terephthalate	45.14	70.76	38.57	62.14	Chen *et al*.^43^
LA/SiO_2_	42.76	47.30	40.12	10.25	Fang *et al*.^44^
LA/SiO_2_	37.50	41.8	33.60	49.6	Yuan *et al*.^45^

## Conclusion

In this research, mesoporous silica nanoscale microspheres (MSNM) was prepared and applied as the supporter of lauric acid (LA) for developing a favorable shape-stabilized PCM (LA/MSNM), and the LA/MSNM was synthesized by a vacuum impregnation method that was simple to operate. On the basis of the research results of characterization tests, the MSNM had high porosity and large surface area, which were beneficial to avoid the leakage of PCM, and its loading capacity for LA was higher than that of the commercially purchased activated carbon. The thermal stability study results demonstrated that the enthalpy of LA/MSNM was higher than that of LA/AC prepared with commercially purchased activated carbon as the supporter of LA. Therefore, LA/MSNM possessed great potential in the practical application of thermal energy storage.

## References

[1] Li M, Wang CC (2019). Preparation and characterization of GO/PEG photo-thermal conversion form-stable composite phase change materials. Renewable Energy.

[2] Chen Y (2019). Efficient Shape-Stabilized Phase-Change Material Based on Novel Mesoporous Carbon Microspheres as a Matrix for Polyethylene Glycol: Preparation and Thermal Properties. JOM.

[3] Wu B (2018). Study on a PEG/epoxy shape-stabilized phase change material: Preparation, thermal properties and thermal storage performance. International Journal of Heat and Mass Transfer.

[4] Li C (2019). Composite phase change materials for thermal energy storage: From molecular modelling based formulation to innovative manufacture. Energy Procedia.

[5] Yi H (2019). Design of MtNS/SA microencapsulated phase change materials for enhancement of thermal energy storage performances: Effect of shell thickness. Solar Energy Materials and Solar Cells.

[6] Gao JK (2019). Facile functionalized mesoporous silica using biomimetic method as new matrix for preparation of shape-stabilized phase-change material with improved enthalpy. International Journal of Energy Research.

[7] Rehmana T, Ali HM, Janjuac MM, Sajjadd U, Yan WM (2019). A critical review on heat transfer augmentation of phase change materials embedded with porous materials/foams. International Journal of Heat and Mass Transfer.

[8] Khattak Z, Ali HM (2019). Air cooled heat sink geometries subjected to forced flow: A critical review. International Journal of Heat and Mass Transfer.

[9] Sajid MU, Ali HM (2019). Recent advances in application of nanofluids in heat transfer devices: A critical review. Renewable and Sustainable Energy Reviews.

[10] Ma LY, Guo CG, Ou RX, Wang QG, Li LP (2019). Synthesis and characterization of the n-butyl palmitate as an organic phase change material. Journal of Thermal Analysis and Calorimetry.

[11] Chen Y (2019). Dopamine functionalization for improving crystallization behaviour of polyethylene glycol in shape-stable phase change material with silica fume as the matrix. Journal of Cleaner Production.

[12] Gao JK (2019). A Facile and Simple Method for Preparation of Novel High-Efficient Form-Stable Phase Change Materials Using Biomimetic-Synthetic Polydopamine Microspheres as a Matrix for Thermal Energy Storage. Polymers.

[13] Wan YC (2019). A promising form-stable phase change material prepared using cost effective pinecone biochar as the matrix of palmitic acid for thermal energy storage. Scientific Reports.

[14] Kuan DY, Dai LG, Liu DH, Du W, Liu HJ (2019). A novel clean process for the combined production of fatty acid ethyl esters (FAEEs) and the ethyl ester of polyunsaturated fatty acids (PUFAs) from microalgae oils. Renewable Energy.

[15] Wan X, Su L, Guo BH (2019). Design and preparation of novel shapeable PEG/SiO_2_/AA shape-stabilized phase change materials based on double-locked network with enhanced heat storage capacity for thermal energy regulation and storage. Powder Technology.

[16] Chen Z, Shan F, Cao L, Fang GY (2012). Synthesis and thermal properties of shape-stabilized lauric acid/activated carbon composites as phase change materials for thermal energy storage. Solar Energy Materials and Solar Cells.

[17] Mehrali M, Latibari ST, Mehrali M, Mahlia TMI, Metselaar HSC (2014). Effect of carbon nanospheres on shape stabilization and thermal behavior of phase change materials for thermal energy storage. Energy Convers.

[18] Weidner MC, Evenson Z, Zamponi M, Possart W (2019). Molecular Motion in Viscous DGEBA with Nanoparticles as Seen by Quasi‐Elastic Neutron Scattering. Macromolecular Chemistry and Physics.

[19] Qian TT (2016). Radial-like mesoporous silica sphere: A promising new candidate of supporting material for storage of low-, middle-, and high-temperature heat. Energy.

[20] Tian F (2017). Thermal properties of nano-sized polyethylene glycol confined in silica gels for latent heat storage. Thermochimica Acta.

[21] Kadoono T, Ogura M (2014). Heat storage properties of organic phase-change materials confined in the nanospace of mesoporous SBA-15 and CMK-3. Physical chemistry chemical physics: PCCP.

[22] Zhang L (2016). phase change materials based on polyethylene glycol supported by graphene-based mesoporous silica sheets. Applied Thermal Engineering.

[23] Jun SH (2012). Highly Efficient Enzyme Immobilization and Stabilization within Meso-Structured Onion-Like Silica for Biodiesel Production. Chemistry of Materials.

[24] Chen Y (2017). Novel shape-stabilized phase change materials composed of polyethylene Glycol/Nonsurfactant-templated mesoporous silica: Preparation and thermal properties. JOM.

[25] Gao JK (2018). High performance shape-stabilized phase change material with nanoflower-like wrinkled mesoporous silica encapsulating polyethylene glycol: preparation and thermal properties. Nanomaterials.

[26] Gao JK, Hou LA, Zhang GH, Gu P (2015). Facile functionalized of SBA-15 via a biomimetic coating and its application in efficient removal of uranium ions from aqueous solution. Journal of Hazardous Materials.

[27] Gao JK, Zhang ZJ, Jiang YJ, Chen Y, Gao SF (2017). Biomimetic-functionalized, tannic acid-templated mesoporous silica as a new support for immobilization of NHase. Molecules.

[28] Chen Y (2018). Preparation and thermal properties of novel shape-stabilized phase change materials based on Polyethylene Glycol/Meso-structured onion-like silica composite. *Science of Advanced*. Materials.

[29] Chen D (2018). Mesoporous silica nanoparticles with wrinkled structure as the matrix of myristic acid for the preparation of a promising new shape-stabilized phase change material via simple method. RSC Adv..

[30] Gao JK (2017). Enhanced Thermal Properties of Novel Latent Heat Thermal Storage Material Through Confinement of Stearic Acid in Meso-Structured Onion-Like Silica. JOM.

[31] Chen Y (2018). Fabrication and characterization of novel shape-stabilized stearic acid composite phase change materials with tannic-acid-templated mesoporous silica nanoparticles for thermal energy storage. RSC Adv..

[32] Gao JK (2019). Dopamine functionalized tannicacid-templated mesoporous silica nanoparticles as a new sorbent for the efficient removal of Cu^2+^ from aqueous solution. Scienfic Reports.

[33] Feng LL, Song P, Yan SC, Wang HB, Wang J (2015). The shape-stabilized phase change materials composed of polyethylene glycol and graphitic carbon nitride matrices. Thermochimica Acta.

[34] Chen Y (2018). Cost-effective biochar produced from agricultural residues and its application for preparation of high performance form-stable phase change material via simple method. International journal of molecular sciences.

[35] Liu DD, Wu ZS, Tian F, Ye BC, Tong YB (2016). Synthesis of N and La co-doped TiO_2_/AC photocatalyst by microwave irradiation for the photocatalytic degradation of naphthalene. Journal of Alloys and Compounds.

[36] Chen Y (2020). A novel strategy for enhancing the thermal conductivity of shapestable phase change materials via carbon-based in situ reduction of metal ions. Journal of Cleaner Production.

[37] O’Neil GW (2018). Alkenones as renewable phase change materials. Renewable Energy.

[38] Faden M, König-Haagen A, Höhlein S, Brüggemann D (2018). An implicit algorithm for melting and settling of phase change material inside macrocapsules. International Journal of Heat & Mass Transfer.

[39] Pielichowska K, Pielichowski K (2014). Phase change materials for thermal energy storage. Progress in Materials Science.

[40] Wei HT, Lia XQ (2017). Preparation and characterization of a lauric-myristic-stearic acid/Al_2_O_3_-loaded expanded vermiculite composite phase change material with enhanced thermal conductivity. Solar Energy Materials & Solar Cells.

[41] Konuklu Y, Ersoyc O, Erzina F, Toramand ÖY (2019). Experimental study on preparation of lauric acid/microwave-modified diatomite phase change material composites. Solar Energy Materials and Solar Cells.

[42] Wen RL (2016). Synthesis and characterization of lauric acid/expanded vermiculite as form-stabilized thermal energy storage materials. Energy and Buildings.

[43] Chen CZ, Wang LG, Huang Y (2008). A novel shape-stabilized PCM: Electrospun ultrafine fibers based on lauric acid/ polyethylene terephthalate composite. Materials Letters.

[44] Fang GY, Li H, Liu X (2010). Preparation and properties of lauric acid/silicon dioxide composites as form-stable phase change materials for thermal energy storage. Materials Chemistry and Physics.

[45] Yuan HM (2019). Size controlled lauric acid/silicon dioxide nanocapsules for thermal energy storage. Solar Energy Materials and Solar Cells.

